# Mast cells in the kidney biopsies of pediatric patients with lupus nephritis

**DOI:** 10.1590/2175-8239-JBN-2018-0222

**Published:** 2020-01-31

**Authors:** Stéfany Silva Santos, Carolina Marques Ramos, Maria Luiza Gonçalves dos Reis Monteiro, Juliana Reis Machado, Marlene Antônia dos Reis, Rosana Rosa Miranda Corrêa, Laura Penna Rocha

**Affiliations:** 1 Universidade Federal do Triângulo Mineiro, Instituto de Ciências Biológicas e Naturais, Departamento de Patologia Genética e Evolução, Setor de Patologia Geral, Uberaba, MG, Brasil.

**Keywords:** Lupus Nephritis, Mast Cells, Pediatrics, Nefrite Lúpica, Mastócitos, Pediatria

## Abstract

**Introduction:**

Mast cells may be involved in inflammation and contribute to the onset of fibrosis in lupus nephritis (LN).

**Objective:**

This study aimed to correlate the presence of mast cells in kidney biopsy specimens of pediatric patients with LN with activity (AI) and chronicity (CI) indices and assess how effectively mast cells may be used as a prognostic factor.

**Method:**

The study included 40 patients aged 6-18 years diagnosed with LN at the Renal Disease Service of the Federal University of Triângulo Mineiro between 1996 and 2015. Workup and epidemiological data were evaluated vis-à-vis AI, CI, and mast cell counts (MCC).

**Results:**

Significant positive correlations were found between mast cell counts (MCC) and AI (*p* = 0.003; r: 0.66) and MCC and CI (*p* = 0.048; r: 0.48). The ROC curve showed that mast cells were highly sensitive and specific in the differentiation of patients with an AI > 12 from individuals with an AI ≤ 12. Serum creatinine levels were higher in individuals with class IV LN than in patients with class V disease [1.50 (0.40-20.90) vs. 0.70 (0.62-0.90), *p* = 0.04]. Blood urea nitrogen had a positive significant correlation with MCC (*p* = 0.002; r: 0.75). A trend toward a negative correlation was observed between MCC and serum albumin (*p* = 0.06; r: -0.5459). Kidney biopsies of patients with nephrotic syndrome had higher MCC [2.12 (0.41-5.140) vs. 0.53 (0.0-3.94), *p* = 0.07].

**Conclusion:**

Inflammatory cell infiltration and morphological differences between cell types in the inflammatory infiltrate are relevant factors in the assessment of the LN. Mast cell analysis and AI/CI assessment may be relevant prognostic indicators for pediatric patients with LN.

## INTRODUCTION

Systemic lupus erythematosus (SLE) is an autoimmune condition triggered by lymphocyte self-tolerance loss, an event that makes them autoreactive and leads to the formation of multiple autoantibodies and potential end-organ damage.^1^^,^ 2 Renal involvement by SLE is called lupus nephritis (LN), a condition known for myriad histopathology variations that may affect all compartments of the kidneys, and a consequently wide array of clinical and morphological manifestations.

The identification of the factors preceding the onset of renal failure and fibrosis is a relevant effort and possibly an important element in defining patient prognosis. Fibrosis develops via pathophysiological mechanisms that act independently from the primary cause of injury, and consists of the excessive accumulation of extracellular matrix replacing normal renal parenchyma.^3^^,^ 4 ^,^ 5 Mast cells contribute to renal fibrosis and mast cell counts have been correlated with the severity of tubulointerstitial injury.^6^

Mast cells are traditionally subdivided into two phenotypes: mast cells secreting tryptase and chymase and mast cells secreting only tryptase. Tryptases are known for their involvement in increased fibroblast (FB) proliferation; increased FB collagen synthesis and IL6 production; increased FB chemotaxis; increased differentiation of FB into myofibroblasts; increased FB contractility; decreased FB apoptosis; activation of epithelial cells and TGFB1; and increased angiogenesis and neutrophil recruitment. The primary functions of chymase granules include conversion of procollagen type 1 into collagen; conversion of angiotensin I into angiotensin II; and protection against fibrosis in ureteral obstruction models. In sum, mast cells secreting tryptase might be involved in immune response, while mast cells secreting chymase might have an additional role in angiogenesis and tissue remodeling.^7^^,^ 8 In the kidneys, mast cells are rarely seen in the glomerular compartment. Mast cells in the renal interstitium are predominantly of the tryptase-secreting type, although the tryptase and chymase-secreting type is found in the tubulointerstitial compartment in some renal conditions, albeit to a lesser degree.^8^

Some of the morphological signs observed in the kidney biopsies of patients with lupus nephritis may be used to predict disease progression and help nephrologists to define the type and intensity of immunosuppressant therapy required. For this reason, authors proposed the segregation of active and chronic disease based on activity (AI) and chronicity (CI) indices.^9^^,^ 10 ^,^ 11

Despite the significant risk of tissue damage, active disease can be reversed and has been associated with better response to immunosuppressant therapy. However, patients with a higher CI are at greater risk of developing chronic kidney disease. Aggressive therapy is thus recommended to individuals with low-to-medium CI so as to maximize the recovery of renal function.^12^

Based on these findings, this study looked into mast cells and interstitial fibrosis in kidney biopsy specimens of pediatric patients with LN and analyzed them for possible correlations with AI and CI. The study also reviewed possible correlations between histopathology, clinical, and workup findings considering patient LN classes assigned based on the examination of kidney biopsy specimens, since few studies have looked at pediatric LN.

## OBJECTIVES

The primary purpose of this study was to assess the role of mast cells in pediatric patients with LN, the associations between mast cells and activity and chronicity indices, and whether mast cells may be seen as a prognostic factor.

## MATERIALS AND METHODS

The Ethics Committee of the Federal University of Triângulo Mineiro approved this study and assigned it certificate no. 1740. The authors collected workup (urine protein, blood urea nitrogen, and creatinine levels) and epidemiological data (age, sex, and skin color). Forty children and adolescents aged 6-18 years submitted to kidney biopsies and diagnosed with lupus nephritis between 1996 and 2015 at the Renal Pathology Service of the Federal University of Triângulo Mineiro, in Uberaba.

Lupus nephritis classification was performed in accordance with the criteria established by the International Society of Nephrology (ISN) and the Renal Pathology Society (RPS) in 2004.

AI and CI were calculated only for individuals with class IV LN, since this was the most prevalent condition on our group. AI is determined based on analysis for endocapillary hypercellularity, glomerular leukocyte infiltration, subendothelial hyaline deposits, and interstitial inflammation. Each finding is graded on a scale of 0 to 3. Fibrinoid necrosis, karyorrhexis, and cellular crescents are also individually analyzed and graded on a scale of 0 to 3. However, since they are more severe, the outcome is multiplied by two. Therefore, AI may range from 0 to 24. CI is based on findings such as glomerulosclerosis, fibrous crescents, interstitial fibrosis, and tubular atrophy. Each finding is graded on a scale of 0 to 3. Therefore, CI may range from 0 to 12.^8^^,^ 9

A renal pathologist examined all the cases included in this study. Mast cells were counted with the aid of Giemsa staining, in which mast cells are stained magenta on account of their ability to show metachromasia.

Mast cells possess peculiar histological traits. They have secreting cytoplasmic granules equipped with the ability to show metachromasia when processed with Romanowsky stains, which include the Giemsa and toluidine blue stains, to name a few. Metachromasia allows the studied histological structure to stain in a different color than the dye. Studies have shown that the Giemsa and toluidine blue stains are equally effective at identifying mast cells in the kidneys. However, the two stains cannot differentiate mast cells secreting tryptase and chymase from mast cells secreting tryptase only.^13^^,^ 14

Mast cells were counted throughout the entire extent of the biopsy specimens and counts were expressed in cells/mm^2^ under 40x magnification (final magnification 1600x). Area calculations were performed with the aid of a stage micrometer measuring 1000 µm, from which the diameter of the field of view of a light microscope was measured. The area of the field was then calculated (A = π x r^2^) and converted to mm^2^.

Picro-sirius red staining was used to characterize interstitial fibrosis. The natural birefringence of collagen-rich areas highlighted under polarized light shows as a reddish color. These areas are marked by an observer with image analysis tool Leica Qwin^®^ on 40x magnification (final magnification 1250x) only in the tubules and interstitium. The entire extent of the biopsy specimens was analyzed. After the area of interest has been defined, the analysis software expresses the outcome as a proportion of the delimited area.

Microsoft Excel was used in statistical analysis. Data analysis was performed on software package GraphPad Prism version 5.00. Quantitative variables were tested for normality with the Kolmogorov-Smirnov test. Variables from the two groups following a normal distribution and with similar variance were compared with Student’s t-test. The results were expressed as mean value ± standard error (X ± SE). Variables from the two groups not following a normal distribution or following a normal distribution with non-similar variances were compared with the Mann-Whitney U test. The results were expressed in terms of median values and minimum-maximum values [Med (Min-Max)]. The correlations between two variables following a non-normal distribution were analyzed via Spearman’s rank correlation coefficient; correlations between two variables following a normal distribution were analyzed via Pearson’s correlation coefficient. The sensitivity and specificity of mast cell counts as a factor in the determination of activity and chronicity indices were verified based on the Receiver Operating Characteristic (ROC) curve. Differences with a calculated probability (p) of less than 5% were deemed statistically significant (*p* < 0.05).

## RESULTS

**Figure 1:**
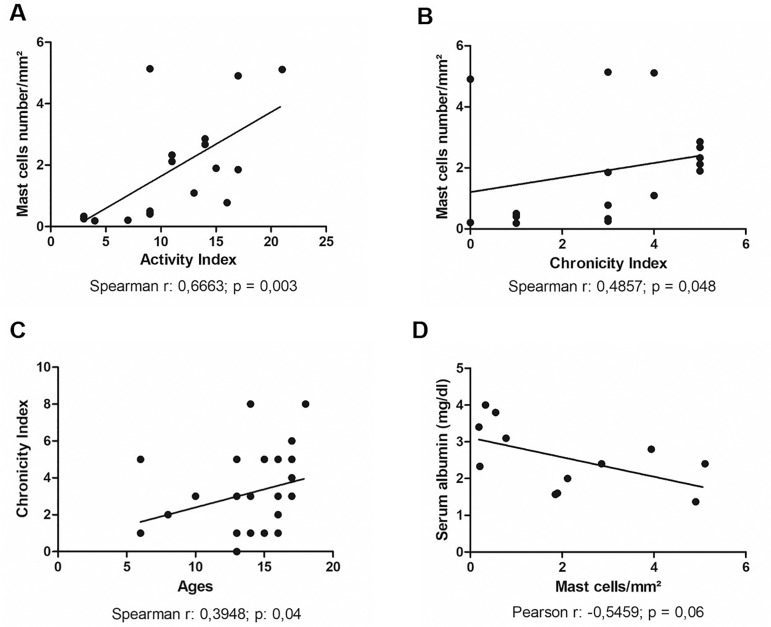
Correlations between morphological and clinical-epidemiological data of pediatric patients with Lupus Nephritis A: Correlation between number of mast cells/mm^2^ and Activity Index B: Correlation between number of mast cells/mm^2^ and Chronicity Index C: Correlation between Chronicity Index and Age D: Correlation between serum albumin and number of mast cells/mm^2^

Forty pediatric patients with a mean age of 14.37 ± 3.30 years (6-18 years) were included in the study. Most (84.61%) were females. In terms of skin color, 71.5% were categorized as whites and 28.5% as non-whites. A significant positive correlation was seen between age and the LN chronicity index (*p* = 0.04; Spearman’s r: 0.3948) (Figure 1C). Table 1 shows clinical and histology data.

**Tabela 1:** Parâmetros clínicos e histológicos dos pacientes pediátricos com nefrite lúpica

	Mediana (mínimo - máximo)
**Proteinúria mg/24hr**	2.000,00 (66,00 - 10.000,00)
**Creatinina mg/dL**	1,41 (0,40 - 8,700)
**Ureia mg/dL**	62,00 (16,00 - 262,00)
**Albumina mg/dL**	2,40 (1,37 - 4,00)
**CKD-EPI Taxa filtração glomerular (mL/min/1.73 m^2^)**	55,30 (6,20 - 166,00)
**Contagem de mastócitos/mm^2^**	
NL Classe IV	0,6650 (0,0 - 5,14)
NL Classe V isolada ou associada a outra classe	0,40 (0,0 - 3,94)

One patient (2.5%) had class I LN; one (2,5%) and class II LN; four (10%) had class III LN; 24 (60%) had class IV LN; two (5%) had isolated class V LN; and six (15%), had class V LN mixed with class III (7.5%) or class IV (7.5%) LN. Therefore, 70% of the biopsies came back with a diagnosis of proliferative (class III or IV) LN, with class IV LN being the most prevalent condition in the study. Biopsies assigned a diagnosis of class IV LN had their activity (AI) and chronicity (CI) indices calculated. The mean AI was 10.58 ± 4.49. The mean CI was 3.26 ± 2.17. Serum creatinine levels were significantly higher in the individuals diagnosed with class IV LN than in the patients with isolated class V LN or mixed class V disease [1.50 (0.40-20.90) *vs.* 0.70 (0.62-0.90), *p* = 0.04].

**Figure 2:**
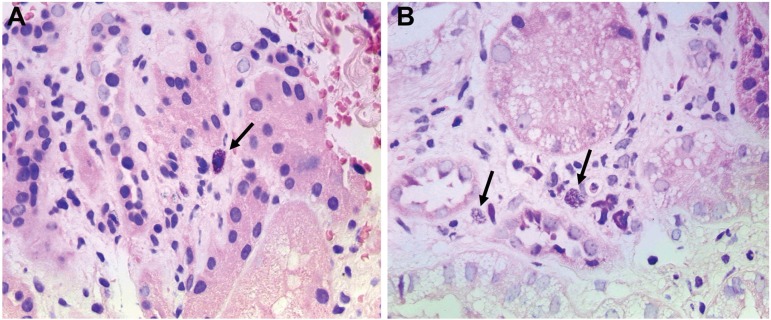
Mast cells of pediatric patients with Lupus Nephritis A: Patient with Lupus Nephritis and activity index less than or equal to 12 B: Patient with Lupus Nephritis and activity index greater than 12

Significant positive correlations were observed between mast cell counts in the tubulointerstitial compartment and the activity index (*p* = 0.003; Spearman’s r: 0.6663) and between mast cell counts in the tubulointerstitial compartment and the chronicity index (p = 0.048; Spearman’s r: 0.4857) (Figures 1A and 1B). We split the patients into groups featuring individuals with an AI > 12, individuals with an AI ≤ 12, individuals with a CI > 4, and individuals with a CI ≤ 4, and subsequently built an ROC curve to verify whether mast cell counts might be used as a sensitive, specific, and accurate marker to differentiate between groups. In the group of patients with an AI > 12, an optimal cutoff set at 0.66 mast cells/mm^2^ had a sensitivity of 100% [95% confidence interval (CI): 63.06% to 100.0%] and a specificity of 80% (95% CI: 51.91% to 95.67%) with an area under the curve of 0.86 and *p* = 0.004 (Figure 2). The group of patients assigned a CI > 4 did not yield statistical significance.

A significant positive correlation was seen between mast cell counts in the tubulointerstitial compartment and blood urea nitrogen levels (*p* = 0.002; Spearman’s r: 0.7538). A trend toward a negative correlation was also observed between mast cell counts in the tubulointerstitial compartment and serum albumin levels (*p* = 0.06; Pearson’s r: -0.5459) (Figure 1D).

Individuals with nephrotic syndrome had higher mast cell counts in the tubulointerstitial compartment than their counterparts without nephrotic syndrome [2.12 (0.41-5.140) *vs.* 0.53 (0.0-3.94), *p* = 0.07].

## DISCUSSION

The mean age of the patients enrolled in our study (14.37 ± 3.30 years) was similar to the mean age of the patients included in other studies, in which ages ranged from 9.6 to 13.6 years.^15^^,^ 16 The variations in mean age probably stem from geographic disparities and differences in inclusion criteria. As expected based on the natural history of the disease, a significant positive correlation was found between age and LN chronicity index, in that chronic injury develops after the disease has progressed for some time.

Most of the individuals included in our study (84.61%) were females, as also reported in other studies enrolling pediatric populations (80% and 81%).^17^^,^ 18 In regard to skin color, 71.5% were categorized as whites. This finding disagrees with current literature, in which approximately 80% of the pediatric patients with SLE are non-whites.^19^ Nevertheless, some studies have also described greater proportions of white patients with LN.^20^^,^ 21 Our study portrayed a region characterized by intense racial mixing, with many significant differences compared with European studies such as one looking into children and adolescents with LN in Italy, in which all included individuals were Caucasians.^21^

In terms of class of disease, our findings showed good agreement with other studies performed with pediatric patients, in which the proportions of subjects with class I LN ranged from 0-0.7%; the proportions of individuals with class II LN ranged from 0-19.3%; the proportions of individuals with class III LN ranged from 17-30.2%; the proportions of individuals with class IV LN ranged from 46.7-70%; the proportions of individuals with isolated class V LN ranged from 10-13.3%; the proportions of individuals with mixed class V LN ranged from 0-18.6%.^16^^,^ 17 ^,^ 21 ^,^ 22 In our study, 70% of the biopsies were consistent with proliferative LN (classes III or IV), as similarly reported in a study carried out in Asia, in which the proportion was 77%.^20^ Universally regarded as the most frequent form of disease, class IV LN was the most prevalent type of disease in our study.^23^

The mean AI was 10.58 ± 4.49, and the mean CI was 3.26 ± 2.17. The mean values found for AI and CI in our study were similar to the values published in an Egyptian study, in which the mean AI was 10.12 and the mean CI 2.06.^24^ Latin American studies including adult patients reported a mean AI of 6.7 ± 4.6 and a mean CI of 2.0 ± 2.7.^25^ These discrepancies may stem from the differences in age of the included patients, since pediatric LN tends to be more aggressive than the disease in adults,^26^ as reflected in the higher chronicity and activity indices.

Creatinine levels were significantly higher in patients with class IV LN than in individuals with isolated or mixed class V LN. A similar study also described higher serum creatinine levels in patients with class IV LN.^27^ Higher serum creatinine levels in proliferative disease may be explained by the pathophysiology of the condition, since subendothelial immune complexes form in class III and IV LN and cause vascular obstruction by endothelial cell edema, leading to decreases in the glomerular filtration rate calculated as a function of serum creatinine.^28^

A significant positive correlation was observed between mast cell counts in the tubulointerstitial compartment and the activity and chronicity indices. The ROC curve also indicated that the presence of mast cells was a sensitive and specific factor to differentiate patients with an AI > 12 from individuals with an AI ≤ 12, a finding suggestive of significant tissue injury and correlated with better chances of responding well to treatment. A similar study also found a correlation between mast cell counts and the two indices.^27^ The correlation found between mast cell counts and the activity index is warranted, since leukocyte infiltration, along with interstitial inflammation, are relevant elements in the activity index. Immune system cell infiltration is the key to the pathogenesis of SLE and include B and T cells, macrophages, dendritic cells, and mast cells.^29^ Activated mast cells can synthesize prostaglandin and leukotriene, which promote the release of cytokines such as tumor necrosis factor alpha, a potent inducer of other inflammatory cytokines, including IL-2 and IL-6. Mast cells, along with their mediators, interact with interstitial infiltration cells, thus contributing to local inflammation.^30^ A recent study also described an important correlation between mast cell counts in the kidneys and CD4, CD8, and CD68 inflammatory cell counts in interstitial infiltrate.^31^ Mast cell chymases and metaloproteinases can convert angiotensin I into angiotensin II, which has been associated with tubulointerstitial inflammation and increased expression of cytokines and growth factors.^32^^,^ 33

The correlation found between mast cell counts in the renal interstitium and the chronicity index is warranted, since interstitial fibrosis is an important element in the chronicity index. As mentioned above, activated mast cells can produce numerous mediators including renin, chymase, tryptase, metaloproteinases (MMP), and transforming growth factor beta (TGF-Beta).^34^ Renin is a substrate for the production of ATII; chymases and MMP-9 can convert ATI into ATII. The local increase in ATII levels promotes fibroblast activation, vasoconstriction, and increased expression of TGF-Beta in the renal interstitium. These events culminate with collagen synthesis and fibrosis.^32^

A significant positive correlation was observed between mast cell counts in the tubulointerstitial compartment and blood urea nitrogen levels, a finding also described in another study looking into primary kidney disease and diabetic nephropathy.^35^ An experimental study described greater mast cell counts in the kidneys of rats with nephropathy induced by a hyperlipid diet, and higher blood urea nitrogen levels compared with controls.^36^ These findings suggest the existence of a close relationship between mast cells in the renal interstitium and blood urea nitrogen.

A trend toward a negative correlation was also seen between mast cell counts in the tubulointerstitial compartment and serum albumin. This finding is apparently unheard of in the literature. We were unable to find other studies in which the same finding has been described for pediatric patients with LN. Differently from our study, the authors of a Chinese study found a positive correlation between mast cell counts in the renal interstitium and serum albumin in adults with anti-glomerular basement membrane glomerulonephritis.^31^ A study enrolling patients with diabetic nephropathy found that serum albumin levels were lower in patients with advanced disease, while mast cell counts in the renal interstitium increased with disease progression.^34^ These disagreements probably stem from the different pathophysiological mechanisms in effect in the studied conditions and reflect the need for additional studies to clarify the involvement of mast cells in renal disease.

After dividing the patients into groups based on presence of absence of nephrotic syndrome, we observed that the patients with nephrotic syndrome had higher mast cell counts in the tubulointerstitial compartment. Pediatric nephrotic syndrome is characterized by the presence of proteinuria, hypoalbuminemia, edema, and hyperlipidemia caused by increased permeability of the glomerular filtration barrier.^37^ Since proteinuria is a key sign of nephrotic syndrome, our findings are in agreement with other studies in which a significant positive correlation was observed between mast cell counts and proteinuria in adults with diabetic nephropathy^34^ or LN.^27^ Mast cells contain chymase and metaloproteinases in their cytoplasmic granules, which play a role in the conversion of ATI into ATII.^32^ Local increases in ATII levels cause increased urine protein levels.^27^ In addition, the association between proteinuria and tubulointerstitial mast cells may warrant the changes in the renal parenchyma subsequent to the onset of proteinuria. Proteinuria is an important risk factor for the development of tubulointerstitial damage, since it promotes the expression of inflammatory and profibrotic mediators that contribute to the influx of mononuclear cells, including mast cells.^38^

## CONCLUSION

Mast cells are involved in acute and chronic disease. The correlation between mast cells and activity and chronicity indices indicate that they have the sensitivity and specificity required to identifying individuals with higher activity indices. Inflammatory cell infiltration and morphological differences between cell types in the inflammatory infiltrate are relevant factors in the assessment of the AI. Mast cells may be an additional tool for renal pathologists to better understand the pathophysiology of lupus nephritis.
